# Layer-by-layer coated silicone-based soft contact lens hydrogel for diclofenac sustained release

**DOI:** 10.1080/07853890.2021.1896898

**Published:** 2021-09-28

**Authors:** Diana Silva, Hermínio C. de Sousa, Maria Helena Gil, Carmen Alvarez-Lorenzo, Benilde Saramago, Ana Paula Serro

**Affiliations:** a CQE, Instituto Superior Técnico, Universidade de Lisboa, Lisboa, Portugal; b CIEPQPF, Departamento de Engenharia Química, Universidade de Coimbra, Coimbra, Portugal; c Departamento de Farmacología, Farmacia y Tecnología Farmacéutica, R + D Pharma Group (GI-1645), Facultad de Farmacia and Health Research Institute of Santiago de Compostela (IDIS), Universidade de Santiago de Compostela, Santiago de Compostela, Spain; d Centro de Investigação Interdisciplinar Egas Moniz (CiiEM), Egas Moniz Cooperativa de Ensino Superior, Caparica, Portugal

## Abstract

**Introduction:**

Soft contact lenses (SCLs) constitute a promising vehicle for ocular drug delivery due to their biocompatibility, prolonged contact with the eye and general acceptance. Soaking in the drug solution is the simplest method to load the drug into the SCLs. However, it usually does not ensure a controlled drug release, compatible with the therapeutic needs. Thus, additional approaches, such as the application of coatings on the SCLs that constitute a barrier to the drug release, have been tried to achieve that purpose [1]. The main goal of this work is to investigate the possibility of using a layer-by-layer (LbL) coating strategy, with alginate, chitosan and hyaluronic acid (ALG/CHI/HA) to get a controlled release of diclofenac from silicon based hydrogels.

**Materials and methods:**

A lab-made silicon-based hydrogel intended for SCLs was loaded by soaking with the anti-inflammatory (diclofenac, DCF) and coated layer-by-layer (LbL) by immersion in solutions of alginate, chitosan and hyaluronic acid. Material properties such as transmittance, wettability, ionic permeability and swelling were studied. Drug release experiments were carried out under sink conditions (3 mL NaCl aqueous solution 130 mM, 36 °C, 180 rpm stirring). The coating stability and lysozyme-coating interaction was evaluated by quartz crystal microbalance with dissipation (QCM-D). A mathematical model was applied to predict the *in vivo* efficacy of the coated lenses. Chorioallantoic membrane (HET-CAM) tests were carried out to predict potential ocular irritation.

**Results:**

The coating did not impair the studied physico-chemical properties, relevant for the application of the material in SCLs. HET-CAM tests did not suggest any potential for ocular irritation of both uncoated and coated samples. QCM-D data revealed the stability of the deposited layers and no adsorption of the protein. DCF release kinetics was controlled by the presence of the coating. The DCF concentration profile in the tear fluid estimated from the mathematical model predicts values above the half maximal inhibitory concentrations (IC_50_) for cyclooxygenase-1 (COX-1) and cyclooxygenase-2 (COX-2) during more than 2 weeks, for the coated hydrogels, while for the uncoated such levels are only expected in the first day of release.

**Discussion and conclusions:**

ALG/CHI/HA LBL coated silicon hydrogels present adequate properties to be used in DCF releasing SCLs. They reveal an antifouling behaviour against lysozyme, one of the most abundant protein in lacrimal fluid. *In vitro* studies suggest that such system has potential to be used in the production of efficient therapeutic SCLs.

## References

[1] Silva D, Sousa HC, Gil MH, et al. Antibacterial layer-by-layer coatings to control drug release from soft contact lenses material. Int J Pharma. 2018;553(1–2):186–200. 10.1016/j.ijpharm.2018.10.041 30342082

